# Improving Antibacterial Activity of a HtrA Protease Inhibitor JO146 against *Helicobacter pylori*: A Novel Approach Using Microfluidics-Engineered PLGA Nanoparticles

**DOI:** 10.3390/pharmaceutics14020348

**Published:** 2022-02-01

**Authors:** Jimin Hwang, Sonya Mros, Allan B. Gamble, Joel D. A. Tyndall, Arlene McDowell

**Affiliations:** 1 School of Pharmacy, University of Otago, Dunedin 9054, New Zealand; hwaji220@student.otago.ac.nz (J.H.); allan.gamble@otago.ac.nz (A.B.G.); joel.tyndall@otago.ac.nz (J.D.A.T.); 2 Department of Microbiology and Immunology, University of Otago, Dunedin 9054, New Zealand; sonya.mros@otago.ac.nz

**Keywords:** *H. pylori*, microfluidics, design of experiments, poly(lactic-*co*-glycolic) acid, size

## Abstract

Nanoparticle drug delivery systems have emerged as a promising strategy for overcoming limitations of antimicrobial drugs such as stability, bioavailability, and insufficient exposure to the hard-to-reach bacterial drug targets. Although size is a vital colloidal feature of nanoparticles that governs biological interactions, the absence of well-defined size control technology has hampered the investigation of optimal nanoparticle size for targeting bacterial cells. Previously, we identified a lead antichlamydial compound JO146 against the high temperature requirement A (HtrA) protease, a promising antibacterial target involved in protein quality control and virulence. Here, we reveal that JO146 was active against *Helicobacter pylori* with a minimum bactericidal concentration of 18.8–75.2 µg/mL. Microfluidic technology using a design of experiments approach was utilized to formulate JO146-loaded poly(lactic-*co*-glycolic) acid nanoparticles and explore the effect of the nanoparticle size on drug delivery. JO146-loaded nanoparticles of three different sizes (90, 150, and 220 nm) were formulated with uniform particle size distribution and drug encapsulation efficiency of up to 25%. In in vitro microdilution inhibition assays, 90 nm nanoparticles improved the minimum bactericidal concentration of JO146 two-fold against *H. pylori* compared to the free drug alone, highlighting that controlled engineering of nanoparticle size is important in drug delivery optimization.

## 1. Introduction

The escalating threat of antibiotic resistance warrants development of antibacterials with a novel mechanism of action and an improved mode of delivery. The majority of antibacterial agents in clinical development share common existing mechanisms of action, targeting bacterial cell wall biosynthesis or deterring protein synthesis on ribosomes, to which multiple resistance mechanisms are already established [1]. There is consensus in the drug design and discovery field that new lead compounds against these existing targets may have already been exhausted and are already exposed to the risk of antibacterial resistance; thus, identification of novel bacterial targets (e.g., bacterial proteases) has been acknowledged as a key approach to mitigate antibacterial insusceptibility [2,3,4,5].

**Figure 1:**
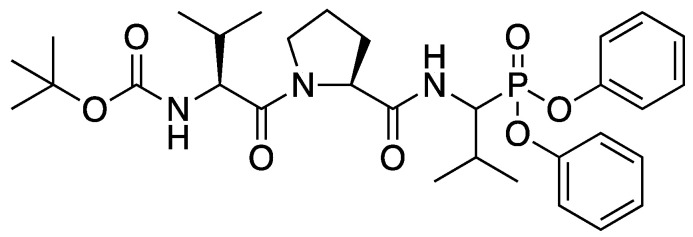
Structure of JO146 initially identified as a lead compound of HtrA protease in *Chlamydia* by high-through screening [6]. JO146 was loaded into PLGA NPs to study the drug delivery to *H. pylori*.

Previously, JO146 (Figure 1) has been identified as a small molecule peptide-based inhibitor of the *Chlamydia trachomatis* high temperature requirement A (HtrA) protease [6]. HtrA is an emerging novel antibacterial target essential in protein quality control and survival under stress conditions [7]. Currently, there are no protease inhibitors used to treat bacterial infections in clinical settings. HtrA is commonly located in the periplasm of Gram-negative bacteria; however, it was demonstrated that *Helicobacter pylori* (*H. pylori*) HtrA is secreted extracellularly and directly contributes to the pathogenesis of the infection [8]. The secreted fraction of *H. pylori* HtrA cleaves E-cadherin, the adherens junction protein in gastric epithelial cells, resulting in disruption of the gastric epithelium and promoting bacterial invasion and paracellular transmigration across host tissues [9]. Although HtrA proteins are evolutionarily well-preserved proteases in bacteria, differences in substrate specificity and mechanistic and/or functional roles in different bacteria are speculated to ascribe JO146 to be a narrow-spectrum antibacterial. JO146 resulted in complete loss of infectious progeny in *Chlamydia* spp. in in vitro and in vivo models, while lacking inhibition against *Staphylococcus aureus*, *Pseudomonas aeruginosa*, and *Escherichia coli* in vitro [10]. In addition, JO146 does not cause toxicity in human, koala, and mice cell lines [6,11]. In the current study, we reveal that JO146 elicits antibacterial activity against *H. pylori*, suggesting that it could be used as an anti-*H. pylori* agent with a novel mechanism of action. Identification of an inhibitor with species-specific activity has become an important strategy of developing antibiotics, which could generate fewer off-target effects on the microbiome and decrease the pressure of antibiotic resistance [5].

Nanoformulations have recently become a way to combat antibacterial resistance and challenges associated with delivery of antibacterial agents, such as low bioavailability, sub-therapeutic drug accumulation in microbial reservoirs, drug-related toxicities, and frequent drug dosing regimens [12]. Nanoparticles (NPs) enable a controlled release of antimicrobials, as well as improving accessibility of the drugs to pathogens and drug targets that are often hard-to-reach due to intracellular localization within bacteria cell membrane barriers containing multiple efflux pumps [13,14]. *H. pylori* infection remains challenging to treat as it mainly colonizes beneath the deep gastric mucosa and adheres to epithelial cells of the stomach [15]. Although *H. pylori* is traditionally viewed as an extracellular Gram-negative bacteria, approximately 1–5% of epithelial cells surrounded by *H. pylori* contain an intracellular population of bacteria, which is implicated in the bacteria’s ability to cause life-long persistence and resistance to antibiotics, particularly those that exhibit poor intracellular accumulation [16,17]. Encapsulation of antimicrobial drugs into NPs can improve muco-adhesion and penetration of cell membranes, as well as intracellular delivery of antibiotics to combat various bacteria (e.g., *Staphylococcus aureus* [18,19,20], *Escherichia coli* [21], *Mycobacterium abscessus* [19], *Klebsiella pneumonia* [22], *Chlamydia trachomatis* [23] and *Mycobacterium tuberculosis* [24,25]), including those that are classified as extracellular pathogens but also show intracellular residence in host cells. Despite its promise as an alternative avenue for combating bacteria, understanding the interaction between NP and cells and management of nanomaterial toxicity often associated with inorganic nanomaterials remain as an important consideration for nanomaterial-based therapeutics [26].

*H. pylori* is one of the most prevalent bacterial pathogens that infects over half the global population and is linked to gastroduodenal diseases, including gastric cancer and peptic ulcers, posing a substantial healthcare burden [27]. *H. pylori* is currently listed as a high priority pathogen by the World Health Organization for research and development of new antibiotics [28]. Currently, triple therapy based on a combination of two antibiotics (clarithromycin plus either amoxicillin or metronidazole) with a proton pump inhibitor is the recommended first-line treatment for *H. pylori* infection [29]. However, *H. pylori* has developed resistance to clarithromycin, amoxicillin, metronidazole, and other antibiotics including levofloxacin, which has resulted in a high rate of treatment failures [30,31,32]. Therefore, new and effective anti-*H. pylori* treatments with a multipronged approach of combining chemical biology and nanoformulation would bring a valuable contribution to the global effort in addressing resistance development.

Based on this initial activity as a novel anti-*H. pylori* agent, we formulated JO146 in poly(lactic-*co*-glycolic) acid (PLGA) NPs to investigate whether the antibacterial efficacy could be enhanced. PLGA NPs are among the most extensively researched drug delivery vehicles due to their outstanding biocompatibility, tuneable degradation features, and a long history of clinical use [33,34]. PLGA NPs have also been utilized to improve drug pharmacokinetics, including low solubility and high sensitivity to chemical and enzymatic degradation [23,24,25]. Hence, these properties may be useful clinically for delivering lipophilic peptide agents such as JO146 (cLogP = 5.60). 

The size of NPs is an important colloidal feature that directly influences cellular uptake, biodistribution, and hence therapeutic efficacy of the particles, determining the in vivo fate of the therapeutic cargo [35,36,37]. A myriad of studies has investigated the influence of particle size on the intracellular uptake in human cells (either normal or cancerous) [36,38,39,40,41]. Although the impact of particle size on the antibacterial activity of metallic NPs has been explored [42,43,44], little is known about their correlation with polymeric NPs. 

Even though size is a critical physicochemical property of NPs that governs bacterial interaction, the lack of well-defined size control technology has limited the exploration of appropriate NP sizing for targeting bacterial cells. The uniformity of particle characteristics, such as size, is significantly affected by the manufacturing processes [45]. Conventional production techniques based on bulk emulsification methods lack the precision, reproducibility, and uniformity over particle size [46]. In recent years, microfluidics technology has emerged as a highly promising technique to produce particles in a highly controlled manner [47,48]. Microfluidics involves mixing of nanolitre scale fluids at high speeds and allows for automation of formulation parameters for production of particles with specific sizes [49,50]. The continuous nature of the microfluidic formulation process is inherently scalable by increasing the amounts of solvents pumped through the system or parallelizing multiple channels and mixers, allowing optimization with minimum requirement of materials that might be scarce or costly [51].

In this study, we report on a combined microfluidics with design of experiments (DoE) approach to produce size-tuneable PLGA particles, which enabled precise assessment of different formulation parameters on physicochemical characteristics of NPs. The DoE approach offers a strategy to make systematic and simultaneous variations in microfluidics settings and rapidly optimize various NP formulations to draw statistical interpretations with a minimum number of experimental runs [52,53,54,55]. This offers a major advantage over traditional “trial and error” approach of changing one variable at a time (OVAT), which is time-consuming and uneconomical. We explored the feasibility of generating different sized nanoparticles with low polydispersity index (PDI) by varying process and formulation parameters such as total flow rate and flow rate ratio of organic and aqueous phases in a DoE approach. We tuned the particle size at sub-100 nm, ~150 nm, and ~220 nm diameter, which represent biologically relevant sizes that influence intracellular particle uptake mechanisms, particle biodistribution, and clearance in vivo [23,35]. Encapsulation efficiency, release profile, and antibacterial efficacy of JO146-loaded PLGA NPs were also assessed.

## 2. Materials and Methods

### 2.1. Materials

PLGA 50:50, ester terminated, MW 15,980 Da, was purchased from Durect Lactel (Cupertino, CA, USA). Acetonitrile (ACN; HPLC grade) was supplied by Merck (Darmstadt, Germany). Mowiol^®^ 4–88 (polyvinyl alcohol, PVA, MW 31,000), phosphate buffered saline (PBS), sucrose (≥99.5%), and tetracycline were purchased from Sigma–Aldrich (St. Louis, MO, USA). Trifluoroacetic acid (TFA, HPLC grade, purity > 99%) was purchased from AK Scientific, Inc. (Union City, CA, USA). Distilled, ultrapure water was obtained from a Milli-Q^®^ Water Millipore Purification System (Billerica, MA, USA). Mueller-Hinton agar (BD Difco, Franklin Lakes, NJ, USA), sheep blood (Fort Richard Laboratories, Auckland, New Zealand), Brucella broth (BD BBL, Franklin Lakes, NJ, USA), foetal bovine serum (FBS; Sigma, Auckland, New Zealand), and tryptic soy agar plates with 5% Sheep blood (Fort Richard Laboratories, Auckland, New Zealand) were utilized to maintain *H. pylori* culture. DMSO (Analytical Reagent Grade) was supplied by Fisher Chemical (Waltham, MA, USA) and MTT by Invitrogen Thermo Fisher Scientific (Waltham, MA, USA). AnaeroPack oxygen absorber-CO_2_ generator was purchased for microaerophilic cultivation from Mitsubishi Gas Chemical (Tokyo, Japan).

### 2.2. Microfluidic Preparation of JO146-PLGA NPs with a Design of Experiments Approach 

JO146 was prepared by in-house synthesis according to our previously reported methods [56]. Two independent sets of D-optimal DoE were conducted using the MODDE GO 12 software (Version 12.0, Umetrics, Umeå, Sweden).

The first DoE aimed at investigating the effects of formulation parameters on NP size (Z-average size) and polydispersity index (PDI). The second DoE was carried out with a goal of determining the lower limit of the PLGA NP size that could be achieved using the microfluidic method. In DoE studies, four input parameters—PLGA concentration (mg/mL), drug concentration (mg/mL), total flow rate (TFR; mL/min), and flow rate ratio (FRR; aq:org *v*/*v*)—were investigated at multilevel settings (low, medium, and high levels, i.e., −1, 0, +1), as described in Table 1 and Table 2. The DoE models were analysed by PLS (partial least squares) regression [57] and one-way ANOVA to evaluate the statistical significance (*p* < 0.05) of the interactions between the input parameters and average particle size and PDI (Tables S1 and S2) [58]. Statistical testing was further conducted for each DoE regression model to obtain value of significance (*p*) and lack of fitness to estimate the error of the model and evaluate the quality of the model by R^2^ and Q^2^ (Table S3) [57,59].

**Figure 2:**
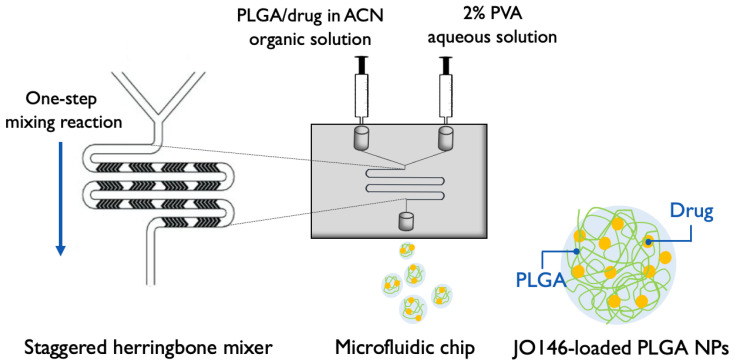
Schematic of microfluidic-assisted NP formulation via nanoprecipitation method using a Y-junction staggered herringbone mixer (modified from [60]). PLGA: poly(lactic-*co*-glycolic acid); ACN: acetonitrile; PVA: polyvinyl alcohol; NP: nanoparticle. Four formulation parameters—TFR, FRR, and polymer and drug concentrations—were investigated in the settings for NP optimization.

PLGA NPs were prepared using a microfluidic staggered herringbone mixer (SHM) with a NanoAssemblr^®^ Benchtop instrument (Precision NanoSystems Inc., Vancouver, BC, Canada). The organic phase containing PLGA and JO146 in ACN and the 2% PVA aqueous phase (*w*/*v*) were injected into the two inlets of the microfluidic cartridge by a syringe pump. The organic and aqueous phases were mixed through the SHM by chaotic advection, leading to nanoprecipitation of JO146-loaded NPs. The formulation parameters (FRR and TFR) of the mixing conditions were programmed into the microfluidic setting according to the DoEs. An initial volume of 0.15–0.25 mL and final volume of 0.05 mL were discarded to collect the NP formulation from the outlet of the cartridge (Figure 2).

### 2.3. Characterization of NPs 

JO146-PLGA NPs were characterized for their size (Z-Average size) and PDI by dynamic light scattering using a Malvern^®^ Zetasizer (Malvern^®^ Nano ZS, Model Zen 3600, Malvern Instruments, Malvern, UK), equipped with a 633 nm laser and 173° detection optics. The zeta potential (ZP) of the NPs were measured by laser Doppler electrophoresis using the same machine but in Malvern^®^ folded capillary zeta cells (Malvern, UK). The NP samples were prepared by diluting 50 µL of the formulations collected from the NanoAssemblr^®^ in 800 µL of ultrapure water and 10 mM NaCl and analysed for characterization in triplicate at 25 °C. Each sample measurement generated an NP count greater than 200 kcps and the derived attenuation factor ranging between 6 and 10. 

### 2.4. Measuring Encapsulation of JO146 in NPs 

Drug encapsulation efficiency (EE) and drug loading (DL) of three selected formulations of different NP sizes (90 nm, 150 nm, and 220 nm) were measured directly from the amount of JO146 entrapped in the NPs using Equations (1) and (2):

(1) $$\begin{array}{l} Encapsulation\;efficiency\;\left(\%\right)\\ =\left(amount\;of\;the\;drug\;in\;the\;pellet\times 100\right)/\left(total\;amount\;of\;the\;drug\;added\right) \end{array}$$

(2) $$\begin{array}{l} Drug\;loading\;\left(\%\right)\\ =\left(amount\;of\;the\;drug\;in\;the\;pellet\times 100\right)/\left(total\;amount\;of\;the\;drug\;and\;PLGA\;added\right) \end{array}$$

The JO146-NP suspensions from the microfluidic preparation were separated in an ultracentrifuge twice, at 45,000 rpm (~138,656× *g*) for 90 nm, 25,000 rpm (~42,795× *g*) for 150 nm, and 15,000 rpm (~15,406× *g*) for 220 nm, at 4 °C for 15 min. Centrifugation speed was optimized for each NP size formulation to facilitate quick re-dispersibility of the pellet, and sufficient centrifugal force was provided to ensure less than 2% loss of NPs in the supernatant. The pellet was dissolved in 2:1:1 DMSO:ACN:H_2_O and sonicated to release the drug from the lysed NPs. The remaining suspension was separated using a centrifuge at 14,500 rpm (14,100× *g*) for 30 min, and the JO146 concentration of the supernatant was analysed by gradient elution using analytical RP-HPLC:deionized water with 0.5% TFA for phase A and ACN with 0.5% TFA for phase B at a flow rate of 1 mL/min (Table 3).

### 2.5. Stability of JO146-PLGA in Simulated Physiological Conditions 

A 2 mL aliquot of each JO146-PLGA NP formulation was prepared as described in the DoE (Table 1 and Table 2). After ultracentrifugation as described in Section 2.4., the pellet from each formulation was re-suspended in 9 mL of PBS (pH 7.4) and divided into three equal aliquots, each of which was transferred to a 7 mL scintillation vial with a magnetic stirrer. The formulations were stirred in closed vials at 100 rpm at 37 °C on a hot plate for 96 h. An aliquot of a formulation sample in each vial was withdrawn, and the NPs were characterized for their size, PDI, and ZP at 0, 12, 24, 48, and 96 h during the stability testing.

### 2.6. Measuring JO146 Solubility by Turbidity and RP-HPLC

The solubility of JO146 was determined at 37 °C in PBS and PBS with 0.5% Tween 80 (*v*/*v*) used as a surfactant. A turbidity assay was used as an initial screening test to determine the range of JO146 solubility. A stock solution of JO146 in DMSO (20 mg/mL) was introduced into the first well containing the aqueous media, leading to drug precipitation. The suspension in the first well was serially diluted from 750 to 100 μg/mL in PBS (pH 7.4) containing 0.5% Tween 80 (*v*/*v*) and 100 to 21.0 μg/mL in PBS alone (pH 7.4). The turbidity of the wells with different JO146 concentrations was measured by a POLARstar^®^ Omega microplate reader (BMG LABTECH, Ortenberg, Germany) in the endpoint mode at the wavelength of 600 nm, at which solubilised JO146 could not be detected. The absorbance of each well was determined by the average of five different measurement points with a 5 s shaking prior to the first measurement. Each concentration level was tested in triplicate. The absorbance readings were normalized by the average reading of the triplicate media controls in the absence of the drug. The solubility range of the drug was defined here as between the concentration that yielded statistically significant increase in the absorbance compared to the media control and the concentration tested one level below.

### 2.7. In Vitro JO146 Release Kinetics

After preparations of 90-nm, 150-nm, and 220-nm NPs, the pellets were re-suspended in 1.5% (g/mL) sucrose in ultrapure water as a cryoprotectant for freeze-drying of NPs. Lyophilisation of NPs was processed by a Labconco^®^ freeze-drier (Kansas City, MO, USA). The NPs were freeze-dried around 0.04 mbar, −30–40°C overnight, and then progressively warmed to room temperature over the next 1–2 days. The lyophilized NPs were suspended in the release media (PBS, pH 7.4, with 0.5% Tween 80, 37 °C) at approximately 1 mg/mL and stirred in closed scintillation vials in triplicate at 100 rpm, 37 °C on a hot plate. For a control group, free JO146 was used. At 0, 0.5, 1, 2, 24, 48, and 96 h time points, 150 μL was sampled and replaced by fresh media to maintain sink conditions. Samples were centrifuged at 14,500 rpm (14,100× *g*) for 15 min at ambient temperature then the supernatants were kept in a −80 °C freezer until analysis using the analytical HPLC to quantify JO146 concentrations. At the end of the release experiment, the remaining NP suspensions were separated using centrifugation and the pellets were dissolved in 2:1:1 DMSO:ACN:H_2_O to determine the concentration of JO146 that was not released. The results of the amount of drug released at pre-determined time points were reported as average values and standard deviations of three independent sets of experiments. 

### 2.8. MIC and MBC of Free JO146 against H. pylori

The minimum inhibitory concentration (MIC) of free JO146 against *H. pylori* (ATCC^®^ 43504) was investigated by a broth microdilution method. A stock solution of 25 mg/mL JO146 in DMSO was diluted in the assay media (Brucella broth with 3% FBS) to give resulting concentrations ranging from 500 µM (300.84 µg/mL) to 1.56 µM (0.94 µg/mL). The highest JO146 concentration tested for the MBC formulation assays was 200 µM (equivalent to 120.34 µg/mL, containing 0.5% DMSO), which was serially diluted by 2-fold to 1.56 µM (0.94 µg/mL) in a 96-well plate (resulting in 100 µL of inhibitor solution in each well). Likewise, a tetracycline control was prepared by adding 100 µL of the drug in the assay media at concentrations ranging from 5.0 to 0.039 µg/mL to the wells. Growth controls without the inhibitor were included in the assays. A 10 µL sample of *H. pylori* broth culture (OD 0.100; final concentration of ~5.0 × 10^5^–10.0 × 10^5^ CFU/mL) was then added to each well, except for the sterility control wells, prior to incubation of the plate for 4–6 days at 37 °C in a microaerophilic environment. To measure MIC, the turbidity of each well was measured by UV spectroscopy at 600 nm. The MIC was defined by the lowest concentration of the inhibitor that resulted in the same average optical density as the negative controls. The results were cross-checked by an MTT colorimetric assay, in which addition of 10 µL of 5 mg/mL MTT dye (3-(4,5-dimethylthiazol-2-yl)-2,5-diphenyltetrazolium bromide) to each well caused a colour change from yellow to pink or light purple in the presence of viable bacteria. Assays were conducted in triplicate and repeated on three independent days with fresh batches of *H. pylori* culture. 

The minimum bactericidal concentration (MBC) of JO146 was measured by taking 10 µL from each well and inoculating onto fresh tryptic soy agar plates with 5% sheep blood. The agar plates were further incubated in a microaerophilic environment at 37 °C for 4–6 days to detect growth of any remaining bacteria. The MBC of each formulation was determined as the minimum concentration required for complete inhibition (as indicated qualitatively by no bacterial growth on the agar plate) from 10 µL of undiluted culture and compared to that of free JO146.

### 2.9. Antibacterial Activity of JO146 and JO146-PLGA NPs against H. pylori

In each of the three independent sets of MBC experiments, a new batch of JO146-PLGA NP formulations was prepared, and each was divided into two aliquots, one of which was used in the antibacterial assays and the other for characterizing the NP size and drug EE%. Tetracycline, free JO146, and empty PLGA NPs were used as controls; a positive growth control (*H. pylori* in assay media without JO146) and negative growth control (assay media only; Brucella broth with 3% FBS and 0.5% DMSO) were included. Freeze-dried JO146-PLGA NPs were re-suspended in the assay media so that the resulting concentration of JO146 entrapped in the NPs was 200 µM. The average concentration of PLGA in the NP resuspensions was 2.8 mg/mL, thus the NP control was also re-suspended at 2.8 mg/mL. Immediately after the re-suspension, 200 µL of each formulation was added to the plate wells in triplicate and serially diluted two-fold down to the eighth concentration (each well containing 100 µL of the NP suspension). Next, 10 µL of *H. pylori* broth culture (OD 0.100 measured at 600 nm UV wavelength; final concentration of ~5.0 × 10^5^–10.0 × 10^5^ CFU/mL) was added to each well, except for the negative growth control wells. The assay plates were incubated for 96 h at 37 °C in microaerophilic environment and shaken at 100 rpm. After the incubation, the assays were inoculated onto fresh tryptic soy agar plates with 5% sheep blood and further incubated to detect growth of any remaining bacteria.

## 3. Results and Discussion

### 3.1. NP Formulation by the Microfluidic-DoE Method and Characterization

A commercially available NanoAssemblr^TM^ cartridge was used in a microfluidic device to optimize NPs with three discrete size categories in a range from sub-100 to 250 nm, which was proposed to be appropriate for endocytic uptake by mammalian host cells, which is required to reach the invasive, intracellular population of *H. pylori*. 

Using the SHM, a set of preliminary experiments was carried out by a traditional OVAT approach, varying one factor at a time for TFR (5, 10, 15 mL/min) and FRR (aq:org 1:1, 2:1, 4:1, 5:1, 6:1 *v*/*v*) at constant concentrations of the polymer and drug to define the approximate range of process parameter settings for the DoE. There was a decreasing trend in the NP size with increasing TFR (Table S4). The NPs also reduced in size as the FRR increased from 1:1 to 5:1, with a small increase in NP size with a change in FRR from 5:1 to 6:1 (Table S4). Taking these preliminary data into consideration, three levels of FRR—1:1, 2:1, and 5:1 (aq:org)—were selected to be investigated for DoE-1.

To systematically assess the SHM-assisted nanoprecipitation method feasibility, a D-optimal design was used to identify the best subset of experiments that provides flexibility in the input parameters [58,61,62]. The first DoE (DoE-1) was used to assess four formulation parameters (concentration of PLGA polymer and drug, FRR, and TFR) in a microfluidic process and investigate the weighted influence of each parameter on the NP size and PDI. Based on the results obtained from DoE-1, the second DoE (DoE-2) was conducted with the aim to investigate the formulation conditions that enabled finding the lower limit of NP size attainable by the PLGA polymer using the NanoAssemblr^®^ microfluidic system. The use of DoE also allowed rational optimization of the experimental conditions to formulate the NP in the target range of sizes with a narrow mean distribution (PDI < 0.3) and high inter-batch reproducibility.

**Figure 3:**
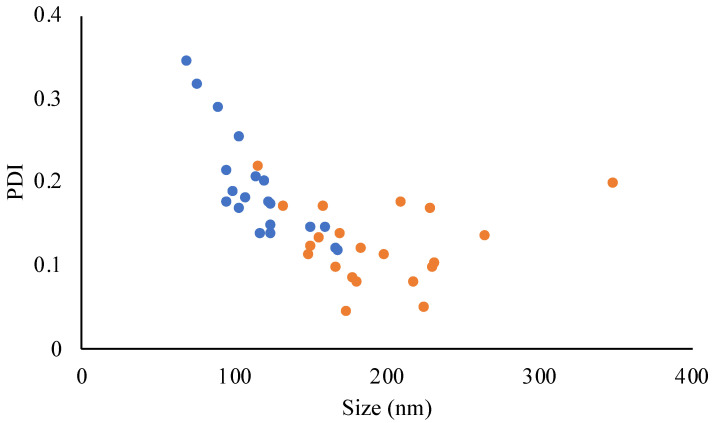
Relationship between the NP size and PDI obtained from DoE-1 (orange) and DoE-2 (blue). Data points are individual measurements.

JO146-PLGA NPs ranging from 70 nm to 349 nm in size were obtained through precise control of the formulation parameters (Tables S5 and S6). All the experiments yielded PDI with <0.3 except experiments no. 14 and 16 in DoE-2, which produced the smallest NP sizes of 70 ± 8.5 and 77 ± 4.3 nm (PDIs of 0.343 and 0.316, respectively; Table S6). The smallest particle size was related to experiments conducted with a high level of TFR (15 mL/min) and FRR (aq:org = 7:1) and a low concentration of PLGA (2 mg/mL). The increase in the PDI with the smallest particle sizes (Figure 3) suggests that when NP size was below 90 nm, the NPs were less stable, hence more prone to aggregation [63,64]. This was supported by the observation that NPs below or close to 80 nm often increased in size by approximately two-fold after ultracentrifugation (data not included). For the purpose of investigating the effect of PLGA NP size on the efficacy of antibacterial delivery, three sizes (90 nm, 150 nm, and 220 nm) were selected (Table 4).

The mean ZP of the NP formulations in DoE-1 and DoE-2 (Tables S5 and S6) was −13 ± 3.78 mV in deionized water and −0.6 ± 0.39 mV in 10 mM NaCl [65]. The ZP of resulting NPs was preserved regardless of the variations in the polymer and drug concentrations, TFR, or FRR. This indicated that chemical properties of the NP surface are maintained despite varying the microfluidic settings.

**Figure 4:**
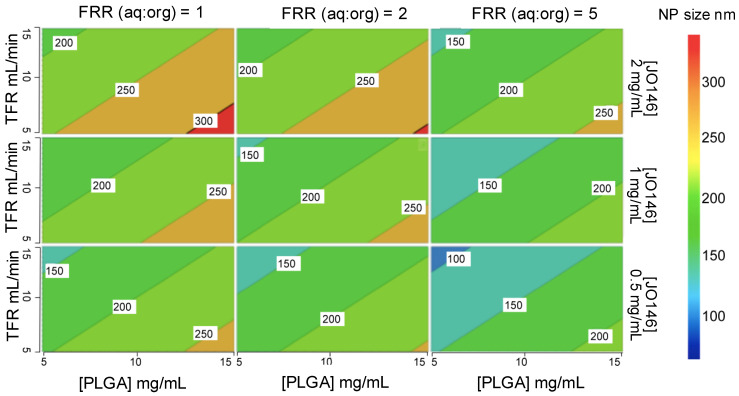
Contour representation of the relationships between the four input parameters and the particle size from DoE-1. TFR: total flow rate; FRR: flow rate ratio.

The significance of each input parameter on the NP size was illustrated by a contour relationship diagram (Figure 4). Particle size is largely influenced by the solvent diffusion coefficient (*D*), mixing time (*t*_mix_), as well as the width of the focused stream (*w_f_*) that is determined by the architectural design of the microfluidic cartridge mixer according to Equation (3) [66]:

*t*_mix_ ≈ *w_f_*^2^/4*D* (3)

Increasing the polymer concentration in the organic solvent phase led to an increase in the NP size (Figure 4). This is ascribed to the higher viscosity of the organic phase with an increased polymer concentration, which resulted in slower diffusion of the organic phase to the aqueous phase, thereby increasing the mixing time [67]. Furthermore, increasing the amount of aqueous phase relative to the organic phase (i.e., higher FRR) accelerated the mixing time, thus decreasing the NP size [68]. In addition, a nucleation mechanism can account for NP formation and growth. Increasing the FRR causes supersaturation of the drug during the mixing process, which leads to an increase in the number of nuclei formed, ultimately decreasing the size of the NPs produced [69]. Additionally, a higher TFR decreased the size of the NPs produced. This is because increasing the speed of solvents pumped into the mixer decreases the size of the droplet formed during the breakup of the organic phase in the aqueous phase, thus the number of nuclei that are formed within the droplet. The decreased number of nuclei within each droplet results in the decrease in the final NP size [69].

Mathematical modelling identified PLGA concentration and FRR as the key variables in the microfluidic process with the highest impact on the NP size in DoE-1 (Table S1). The order of the most influential parameters on the NP size in DoE-1 based on the normalized regression coefficients was found to be PLGA concentration (0.53) > FRR (−0.50) > TFR (−0.42) > drug concentration (0.31; Table S1). In comparison, the influence of each parameter in DoE-2 had a similar but more distinctive ranking: PLGA concentration (0.73) >> TFR (−0.58) >> FRR (−0.26, n.s., *p* > 0.05) >> drug concentration (0.04, n.s., *p* > 0.05; Table S2). The fact that FRR showed a statistically not significant (n.s.) influence on size in DoE-2 could be interpreted that once the volume difference between the aqueous and organic phases exceeds a certain threshold (close to aq:org 5:1 as observed in the preliminary study using the OVAT approach), the correlation between the size and FRR becomes less conspicuous. This could be due to an excessive pressure difference between the aqueous and organic phases flowing into the microchannel, resulting in a backflow of the solvents into the organic inlet. Only FRR conferred significant change in PDI in both DoEs with increasing FRR (aq:org) giving a lower PDI, hence a narrower distribution of size (Tables S1 and S2).

The calculated values of R^2^ and Q^2^ in the DoEs were all above the recommended values from the literature (R^2^ > 0.75, Q^2^ > 0.60) [70], indicating that the model generated in each DoE showed high accuracy in fitting the existing data and predicting future data, respectively (Table S3). In addition, the difference between R^2^ and Q^2^ was below 0.3, suggesting that a high R^2^ value was not due to over-fitting of the data between the parameters and responses [71,72]. These results reinforce the ability of the microfluidic technology to precisely control the input parameters and therefore produce NPs with corresponding characteristics with low variability and high reproducibility. 

### 3.2. JO146-PLGA Stability 

**Figure 5:**
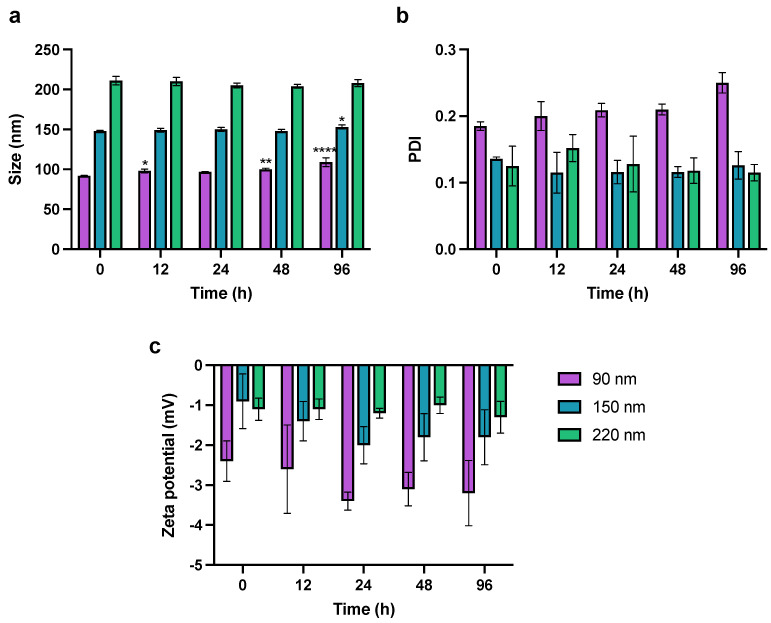
Monitoring of change in NP (**a**) size, (**b**) PDI, and (**c**) zeta potential in PBS at pH 7.4, 37 °C over 96 h. Values represent the mean ± SD (* *p* ≤ 0.05, ** *p* ≤ 0.01, **** *p* ≤ 0.001; one-way ANOVA; Dunnett’s multiple comparisons test).

The physical stability of PLGA NPs was assessed in vitro in PBS (pH 7.4) at 37 °C for 96 h. The evaluation of NP stability was essential to ensure that the in vitro drug release kinetics were not influenced by drug leakage or dumping due to NP instability or degradation. The 90-nm NPs increased in size by 20% over 96 h. The increase in size of 90-nm NPs was statistically significant after 12 h (98 ± 2.4 nm), 48 h (100 ± 1.4 nm) and 96 h (109 ± 5.5 nm) compared to the initial size at 0 h (Figure 5). The size of the 150-nm NPs increased only slightly from 148 to 153 nm after 96 h (*p* ≤ 0.05), and the 220-nm NPs remained the same size over the whole incubation period (one-way ANOVA analysis; Dunnett’s multiple comparisons test). This size-dependent stability of NPs could be explained by the swelling mechanism of the PLGA polymeric matrix as the water penetrates inside the NPs during the bulk diffusion process [73,74]. Small NPs with a high surface to volume ratio could be more susceptible to hydrolytic degradation and physical instability as suggested by higher PDI and aggregation during ultracentrifugation as discussed above. This could be further supported by the complementary increase in PDI as the 90-nm NP size increases throughout the incubation time. Despite this, given that the PDI remained below 0.3 after 96 h, 90-nm NPs were regarded as monodisperse and relatively stable, suitable to deliver JO146 during the incubation of the drug treatment with *H. pylori* in in vitro antibacterial assays [75]. In addition, the ZP of the NPs remained constant regardless of their size at all sampling time points. The stability results of JO146-PLGA NPs showed that low ZP of the NPs does not necessarily reflect low stability of the NPs.

### 3.3. In Vitro Release Kinetics

Release kinetics of JO146 from three different sizes of PLGA NPs were investigated using in vitro studies in PBS (pH 7.4) containing 0.5% Tween 80. Due to the poor aqueous solubility of JO146, 0.5% Tween 80 was utilized as a surfactant to allow JO146 to remain in solution for subsequent quantification using analytical HPLC. The solubility range of JO146 in PBS alone and PBS containing 0.5% (*v*/*v*) Tween 80 was initially screened by turbidity assays [76]. The solubility of JO146 in PBS alone and PBS containing 0.5% Tween 80 (*v*/*v*) were determined to lie in the range of 64–80 μg/mL and 237–316 μg/mL, respectively (Figure S2). This showed that adding 0.5%. Tween 80 (*v*/*v*) increased the aqueous solubility of JO146 in PBS, which aids the investigation of the drug release kinetics.

**Figure 6:**
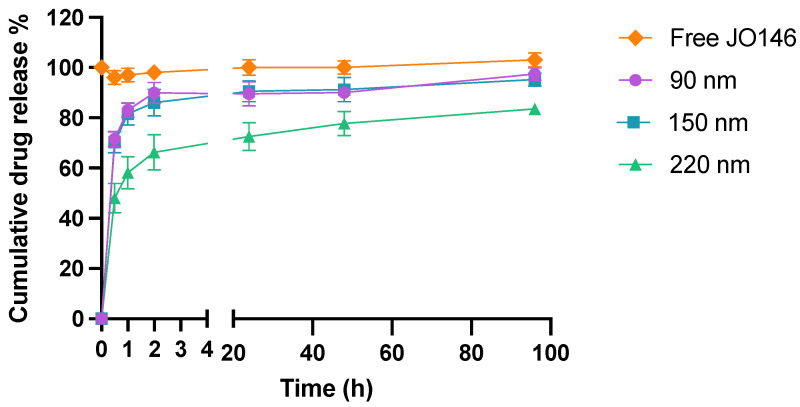
In vitro drug release kinetics of JO146 in PBS (pH 7.4, 37 °C) with 0.5% Tween 80 (*n* = 3). Error bars represent standard error of the mean (SEM).

The release of the drug from NPs was biphasic with a burst where the majority of drug release occurs over the first 2 h followed by on onset of slower drug release up to 96 h (Figure 6). The 90-nm and 150-nm NPs demonstrated very similar first-order drug release kinetics with a release of approximately 80% of the entrapped drug within the first hour (R^2^ = 0.851 and 0.852 for 90 and 150 nm, respectively). In comparison, 220-nm NPs released less than 50% during the first half an hour (R^2^ = 0.876), which was about 1.5-fold less than the smaller NPs. This result aligns well with the theory that smaller NPs have a higher surface to volume ratio, hence facilitating greater release of drug in the initial burst release phase [77]. The second phase of release was zero order, unlike 90-nm 150-nm NPs, which released up to 97% and 95% of the drug after 96 h (R^2^ = 0.803 and 0.914, for 90 and 150 nm, respectively); 220-nm NPs exhibited released up to 84% by the end of the experiment (R^2^ = 0.954) (Figure 6). The average concentration of the free JO146 in the control group was constant throughout the 96-h experiment, confirming that the quantified JO146 concentrations accurately reflect the amount released from the NPs at each time point because JO146 did not degrade under the release conditions.

### 3.4. Antimicrobial Activities of Free and Formulated JO146 against H. pylori

HtrA is crucial for the growth of *H. pylori* and recognized as an important virulence factor attributed to its ability to degrade E-cadherin-based adherens junctions, which allows efficient bacterial transmission during infections [78]. Previously, small-molecule inhibitors have been identified by a series of virtual screening and fragment-based, *de novo* design strategies to target the *H. pylori* HtrA serine protease [79], corroborating that HpHtrA protease can be targeted by small molecules. However, no peptide-based or substrate-based protease inhibitors have yet been reported against HpHtrA.

Three independent MIC assays were conducted with tetracycline as a positive control, which inhibited *H. pylori* with MIC and MBC values of 0.63–1.25 µg/mL and 0.625–2.5 µg/mL, respectively (Table S7). The variability in the inhibitory concentrations was consistent with values reported in the literature for *H. pylori* [80,81]. JO146 exhibited moderately strong antibacterial activity against *H. pylori* with a MIC range of 50–62.5 µM (30–37.6 µg/mL), which is a promising level of inhibition, comparable to the antibacterial activity against *C. trachomatis* [6]. 

Despite a lack of antibacterial activity against *E. coli*, *P. aeruginosa*, and *S. aureus* [6,10], the ability of JO146 to inhibit the growth of *H. pylori* could indicate that *H. pylori* HtrA has more similar substrate specificity to CtHtrA than those in other bacteria [82]. 

The MBC of JO146 was determined to be 31.3–125 µM (18.8–75.2 µg/mL), which was within the range of the MIC reported above, indicating that the drug likely acts via a bactericidal mechanism rather than being bacteriostatic [83]. This was not unexpected given that JO146 is a covalent transition state analogue that irreversibly binds to the biological target. Haemolysis of the sheep blood cells was not observed with any concentrations of JO146 tested. To further validate the bactericidal activity of JO146, it would be necessary to perform bacterial-killing kinetics assays in subsequent studies.

**Figure 7:**
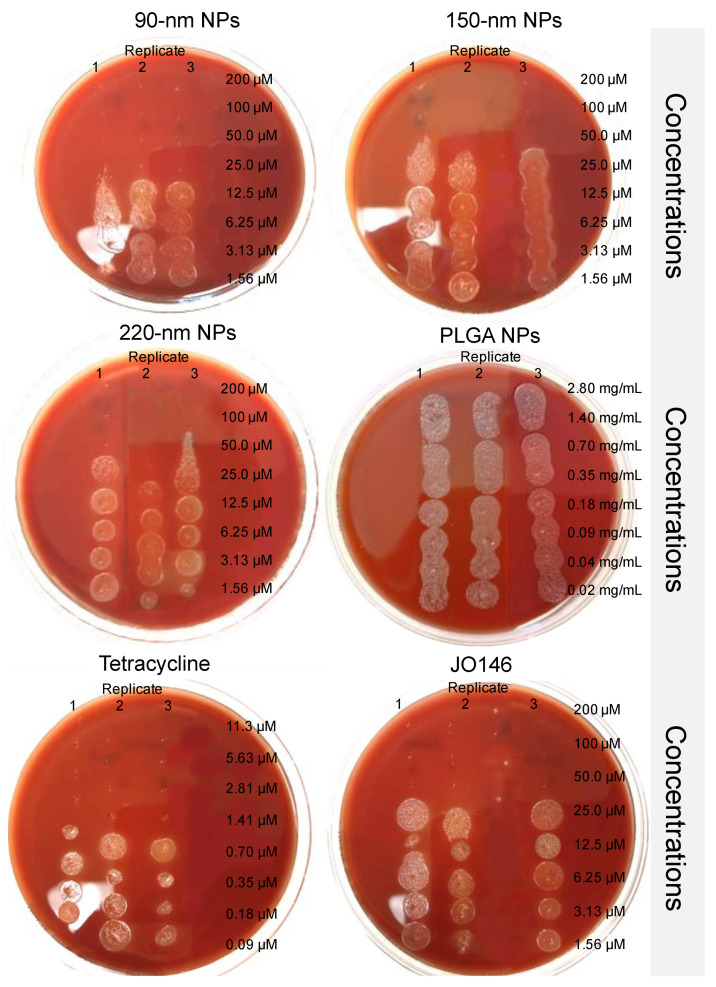
Minimum bactericidal concentration assays conducted to determine viability of *H. pylori* treated with JO146-PLGA NPs, drug-free PLGA NPs, tetracycline, and JO146. The efficacy of JO146 formulated in PLGA NPs was determined as MBC required to kill *H. pylori* (i.e., no visible growth of bacterial colonies). MBC assays were conducted in triplicate on tryptic soy agar plates with 5% sheep blood.

The 150-nm and 220-nm NPs did not affect the MBC of JO146 (50 μM) compared to that of free JO146 (Figure 7). However, the MBC of JO146 delivered by 90-nm NPs improved two-fold (25 μM). This was despite the finding that 90-nm and 150-nm NPs showed similar drug release kinetics, indicating that the difference in the efficacy of JO146 delivered by the two NP formulations could be attributed to the NP size. In comparison to mammalian cells that can endocytose NPs up to several hundred nanometres, bacteria can have very limited uptake of NPs due to their cell wall forming a barrier against simple diffusion and the lack of facilitated uptake such as endocytosis [84,85]. However, NPs may interact with bacteria by different mechanisms: (1) fusion with the microbial cell wall or cell membrane, transporting the antibacterial agent within the membrane barrier [86], (2) adsorption to the cell wall surface and continuous release of the antibacterial agent [86], and (3) alteration of the bacterial membrane potential to enhance membrane permeability [87]. Although 220-nm NPs displayed more sustained release (releasing ~80% over 96 h) compared to 90-nm and 150-nm NPs, the rate of drug release did not seem to affect the overall viability of *H. pylori* in vitro after the 96-h incubation. However, NP size had greater influence on the bacterial growth, suggesting that the superior surface-to-volume ratio of 90-nm NPs allowed greater interaction with the bacterial cell wall [37,88], thereby increased drug exposure to the periplasmic target site where HtrA proteins are commonly localized in Gram-negative bacteria [7]. Future research against an intracellular population of *H. pylori* and in vivo testing of the formulations would provide insight on the clinical benefits of utilizing a highly defined distribution of NP sizes for the treatment of *H. pylori* infections that are resistant to traditional antibiotics.

## 4. Conclusions

Although PLGA NPs have been extensively studied with a wide range of therapeutics, the optimal NP size for targeting bacteria is unclear, and the optimization of the NPs using the traditional bulk mixing methodology remains challenging.

In this paper, a combined microfluidics with DoE approach enabled assessment of the four key formulation parameters (drug and polymer concentrations, TFF, FRR) on physicochemical characteristics of NPs. Three different sized JO146-PLGA NPs (90-, 150-, and 220-nm) were formulated with a narrow distribution of size to investigate the optimal NP size to facilitate improved delivery of JO146 to *H. pylori*. These results showed the NanoAssemblr^®^ microfluidic platform enabled precise control of the parameters to fine-tune physicochemical properties of NPs that might be required for specific clinical applications. NP size significantly influenced the particle stability and drug release kinetics, and 90-nm NPs improved MBC against *H. pylori* two-fold compared to 150-nm and 220-nm NPs and free JO146 in in vitro antibacterial assays, indicating that NP size plays a critical role in drug delivery and enhancing the antibacterial potency of the encapsulated drug. In vivo study of the antibacterial activity of the NP formulations compared to JO146 is required to investigate the biological and clinical significance of the size-controlled NPs.

## Figures and Tables

**Table 1:** Input parameters of DoE-1 for the formulation of JO146-PLGA NPs with sizes in the range of 110–270 nm. Total flow rate = 5, 10, 15 mL/min and flow rate ratio = 1, 2, 5.

Parameters	−1	0	+1
PLGA concentration (mg/mL)	5	10	15
TFR (mL/min)	5	10	15
FRR (aqueous:organic)	1	2	5
	0.5	1	2

**Table 2:** Input parameters of DoE-2 for the formulation of JO146-PLGA NPs with size targeted below 100 nm. Total flow rate = 10, 15 mL/min and flow rate ratio = 3, 5, 7.

Parameters	−1	0	+1
PLGA concentration (mg/mL)	2	4	6
TFR (mL/min)	10	-	15
FRR (aqueous:organic)	3	5	7
	0.5	-	1

**Table 3:** Gradient method for the analytical RP-HPLC used for JO146 quantification.

Time (min)	Phase A%	Phase B%
1.00	40	60
10.00	15	85
11.00	40	60
15.00	40	60

**Table 4:** Three selected sizes of JO146-PLGA NPs from the design of experiments analysis.

Size (nm)	[PLGA] (mg/mL)	FRR (aq:org)	TFR (mL/min)	[JO146] (mg/mL)	PDI	ZP (mv)	EE%	DL%
90	10	1	10	1	0.096	−9.6 ± 2.0	5	1.3
150	5	5	10	2	0.170	−8.0 ± 2.0	16	1.6
220	2	3	15	0.5	0.173	−13.8 ± 0.4	25	3.9

## Data Availability

Data available on request.

## Supplementary Materials

The following are available online at https://www.mdpi.com/article/10.3390/pharmaceutics14020348/s1. Table S1: Normalized regression coefficients of DoE-1, Table S2: Normalized regression coefficients of DoE-2, Table S3: Diagnostic parameters of DoE model validity, Table S4: OVAT investigation of the effects of TFR and FRR on NP size, Table S5: Experimental conditions of DoE-1 and JO146-PLGA nanoparticle characterization, Table S6: Experimental conditions of DoE-2 and JO146-PLGA nanoparticle characterization, Table S7: Quantitative and qualitative estimation of MIC of free JO146 by MTT colorimetric assays. Figure S1: DoE-2 contour plot of the influence of the input parameters on NP size, Figure S2: JO146 solubility determination by turbidity assays.
