# Supplementary material for: Improving Antibacterial Activity of a HtrA Protease Inhibitor JO146 against Helicobacter pylori: A Novel Approach Using Microfluidics-Engineered PLGA Nanoparticles
Source: Pharmaceutics. 2022 Feb 1;14(2):348. doi: 10.3390/pharmaceutics14020348 (PMC8875321; doi:10.3390/pharmaceutics14020348)
Supplement: Supplementary file 1 [file pharmaceutics-14-00348-s001.zip › pharmaceutics-1522260-supplementary.pdf]

pharmaceutics

Supplementary Materials: Improving Antibacterial Activity of a HtrA Protease Inhibitor JO146 against *Helicobacter pylori*: A Novel Approach Using Microfluidics-Engineered PLGA Nanoparticles

Jimin Hwang, Sonya Mros, Allan B. Gamble, Joel D. A. Tyndall and Arlene McDowell

Supplementary Material S1. Normalized regression coefficients of DoEs to investigate the significance of each experimental parameter on the nanoparticle characterization measurements

The regression coefficients derived from the MODDE software are used to assess if model parameters are significantly different from zero (null hypothesis significance tests) [1]. The displayed values are computed from factor values scaled to unit variance then normalized with respect to the standard deviation of their respective response to compare between responses.

Table S1. Normalized regression coefficients of DoE-1.

Responses	[PLGA]	[JO146]	FRR	TFR
Size	0.53	0.31	-0.50	-0.42
PDI	0.20[ns]	0.18[ns]	0.67	0.01[ns]

ns= statistically not significant (p >0.05); one-way ANOVA analysis using MODDE GO 12 software.

Table S2. Normalized regression coefficients of DoE-2.

Responses	[PLGA]	[JO146]	FRR	TFR
Size	0.73	0.04[ns]	-0.26[ns]	-0.58
PDI	-0.68	-0.04[ns]	0.56	0.22[ns]

ns= statistically not significant (p >0.05); one-way ANOVA analysis using MODDE GO 12 software.

Supplementary Material S2: Summary of results for the statistical analysis of the NP size for the microfluidic method in DoE-1.

Statistical analysis was conducted by one-way ANOVA using MODDE GO 12 software [1]. Regression analysis and model validity were done by R^2 and Q^2 diagnostic tools [2]. R^2 represents the goodness of fit, which measures how well the regression model fits the raw data. Q^2 indicates the goodness of prediction, which estimates the predictive power of the model. Model validity larger than 0.25 represents that the model has no lack of fit [1]. This indicates that the model error is not significantly larger than the pure error. Reproducibility is the variation of the response (often at the centre point) under the same conditions as the total variation of the response. These diagnostic parameters of 1 signify perfect models. Statistical testing was further conducted for each DoE regression model to obtain value of significance (p) and lack of fitness to estimate the error of the model.

Table S3. Diagnostic parameters of DoE model validity.

DoE	R^2	Q^2	Model validity	reproducibility	Regression (p)	Lack of fitness
DoE-1	0.790	0.655	0.4	1.00	0.00006	0.084
DoE-2	0.934	0.836	0.6	0.934	<0.0005	0.194

Supplementary Material S3: Preliminary study of the relationship between TFR and FRR (inputs) and nanoparticle size (response) produced by the microfluidic device using the traditional OVAT approach. TFR: total flow rate; FRR: flow rate ratio.

Table S4. OVAT investigation of the effects of TFR and FRR on NP size.

Run No.	PLGA concentration (mg/mL)	Drug concentration (mg/mL)	TFR (mL/min)	FRR (aq:org)	Size (nm)
1	10	1	5	1	250
2	10	1	5	2	216
3	10	1	5	4	208
4	10	1	5	5	191
5	10	1	5	6	209
6	10	1	10	1	247
7	10	1	10	2	191
8	10	1	10	4	180
9	10	1	10	5	168
10	10	1	10	6	175
11	10	1	15	1	234
12	10	1	15	2	172
13	10	1	15	4	175
14	10	1	15	5	150
15	10	1	15	6	156

Supplementary Material S4: DoE input parameters and JO146-PLGA nanoparticle characterization of size, PDI and zeta potential.

Table S5. Experimental conditions of DoE-1 and JO146-PLGA nanoparticle characterization.

No.	[PLGA] (mg/mL)	TFR (mL/min)	FRR (aq:org)	[JO146] (mg/mL)	Size ± SD (nm)	PDI	ZP ± SD (mV)[a] (10 mM NaCl)
1	5	5	5	0.5	156 ± 1.0	0.132	-9.3 ± 0.7 (-0.7 ± 0.14)
2	15	5	5	0.5	211 ± 1.4	0.173	-19.6 ± 0.2 (-0.6 ± 0.08)
3	10	15	5	0.5	133 ± 2.0	0.169	-8.8 ± 0.9 (-0.6 ± 0.08)
4	10	5	1	0.5	218 ± 2.0	0.079	-11.5 ± 1.3 (-0.8 ± 0.07)
5	5	15	1	0.5	174 ± 1.5	0.042	-8.6 ± 0.7 (-1.0 ± 0.12)
6	15	15	1	0.5	233 ± 2.3	0.101	-18.2 ± 0.4 (-0.6 ± 0.11)
7	5	10	2	0.5	150 ± 1.5	0.111	-8.9 ± 0.5 (-0.8 ± 0.16)
8	10	5	5	2	229 ± 2.7	0.167	-16.5 ± 0.6 (-0.8 ± 0.17)
9	15	15	5	2	171 ± 2.5	0.135	-19.4 ± 0.4 (-0.8 ± 0.03)
10[b]	5	10	5	2	154 ± 2.6	0.170	-8.0 ± 2.0 (-0.8 ± 0.20)
11	5	5	1	2	225 ± 2.0	0.047	-8.7 ± 0.4 (-0.7 ± 0.15)
12	15	5	1	2	349 ± 5.9	0.196	-19.9 ± 0.2 (-0.6 ± 0.06)
13	10	15	1	2	266 ± 4.9	0.134	-15.5 ± 0.6 (-2.9 ± 0.08)
14	5	15	2	2	151 ± 2.2	0.122	-8.0 ± 0.6 (-0.9 ± 0.14)
15	15	15	2	2	199 ± 2.7	0.110	-20.3 ± 1.4 (-0.8 ± 0.23)
16	5	15	5	1	117 ± 2.4	0.216	-9.3 ± 1.8 (-1.3 ± 0.35)
17[b]	10	10	1	1	225 ± 2.5	0.096	-13.8 ± 0.4 (-0.7 ± 0.22)
18	5	5	2	1	167 ± 1.4	0.096	-8.2 ± 1.4 (-0.8 ± 0.12)
19*	10	10	2	1	179 ± 1.7	0.084	-17.2 ± 0.5 (-0.9 ± 0.21)
20*	10	10	2	1	184 ± 2.1	0.119	-17.2 ± 0.3 (-0.8 ± 0.14)
21*	10	10	2	1	182 ± 0.7	0.077	-17.8 ± 0.3 (-0.8 ± 0.19)

[a]ZP measured in ultrapure distilled water; [b]Selected formulation(s) for JO146 carrier; *Centre point run (experimented in triplicate).

Table S6. Experimental conditions of DoE-2 and JO146-PLGA nanoparticle characterization.

No.	[PLGA] (mg/mL)	TFR (mL/min)	FRR (aq:org)	[JO146] (mg/mL)	Size ± SD (nm)	PDI	ZP ± SD (mV)[a] (10 mM NaCl)
1	2	10	3	0.5	121 ± 2.4	0.200	-15.1 ± 2.4 (-1.4 ± 0.56)
2	6	10	3	0.5	168 ± 3.5	0.117	-16.9 ± 0.8 (-0.9 ± 0.22)
3	2	10	3	1	125 ± 1.7	0.146	-13.4 ± 1.3 (-1.1 ± 0.24)
4	6	10	3	1	169 ± 1.6	0.115	-15.3 ± 1.5 (-0.7 ± 0.07)
5	2	10	7	0.5	104 ± 3.2	0.253	-12.0 ± 2.7 (-0.8 ± 0.18)
6	6	10	7	0.5	151 ± 2.1	0.143	-15.2 ± 1.6 (-0.7 ± 0.09)
7	2	10	7	1	90 ± 1.2	0.288	-14.0 ± 4.6 (-1.1 ± 0.26)
8	6	10	7	1	161 ± 4.0	0.143	-14.0 ± 0.6 (-0.7 ± 0.14)
9	4	15	5	0.5	125 ± 1.7	0.172	-11.1 ± 0.7 (-1.2 ± 0.66)
10[b]	2	15	3	0.5	94 ± 1.2	0.173	-9.6 ± 2.0 (-1.0 ± 0.24)
11	6	15	3	0.5	118 ± 2.9	0.137	-13.9 ± 1.4 (-0.6 ± 0.19)
12	2	15	3	1	96 ± 1.0	0.211	-11.0 ± 1.8 (-1.2 ± 0.36)
13	6	15	3	1	125 ± 2.6	0.136	-13.5 ± 0.6 (-0.7 ± 0.07)
14[c]	2	15	7	0.5	70 ± 8.5	0.343	-8.5 ± 1.1 (-1.2 ± 0.59)
15	6	15	7	0.5	123 ± 1.3	0.174	-13.5 ± 3.5 (-0.9 ± 0.06)
16[c]	2	15	7	1	77 ± 4.3	0.316	-11.1 ± 2.1 (-1.4 ± 0.28)
17	6	15	7	1	116 ± 1.6	0.203	-10.7 ± 0.2 (-0.6 ± 0.13)
18*	4	15	5	1	100 ± 1.9	0.187	-8.6 ± 1.3 (-0.8 ± 0.08)
19*	4	15	5	1	104 ± 3.8	0.166	-11.8 ± 1.6 (-0.7 ± 0.08)
20*	4	15	5	1	108 ± 1.1	0.178	-12.3 ± 1.1 (-0.8 ± 0.13)

[a]ZP measured in ultrapure distilled water; [b]Selected formulation for JO146 carrier; [c]PDI > 0.3; *Centre point run (experimented in triplicate).

Supplementary Material S5: Contour representation of the relationships between the four input parameters and the particle size from DoE-2. TFR: total flow rate; FRR: flow rate ratio

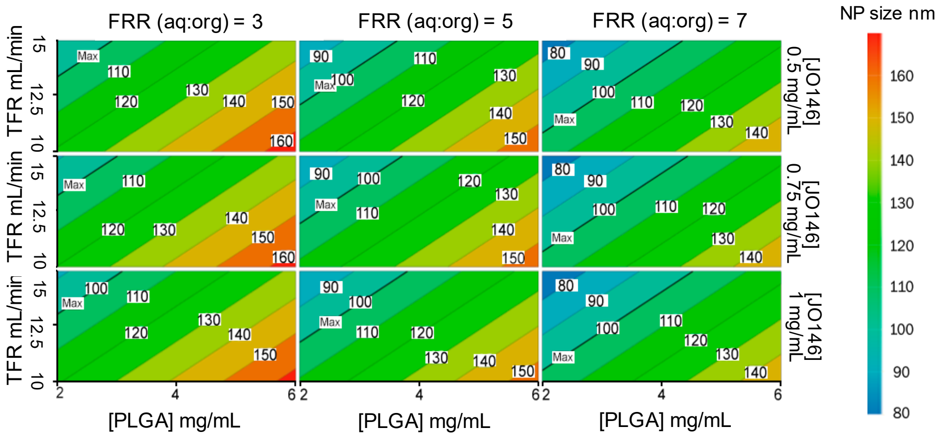

Figure S1. DoE-2 contour diagram of the influence of the input parameters on NP size.

Supplementary Material S6: Contour representation of the relationships between the four input parameters and the particle size from DoE-2. TFR: total flow rate; FRR: flow rate ratio

Turbidity of JO146 dissolved in (a) PBS alone and (b) PBS with 0.5% Tween 80 measured by the increase in normalized absorbance (± SEM) at 600 nm compared to media

control. The solubility of JO146 in PBS alone and PBS containing 0.5% Tween 80 (v/v) was determined 64-80 µg/mL and 237-316 µg/mL, respectively. Statistical significance was determined by one-way ANOVA, *p <0.05; ****p <0.0001 (Dunnett's multiple comparisons test).

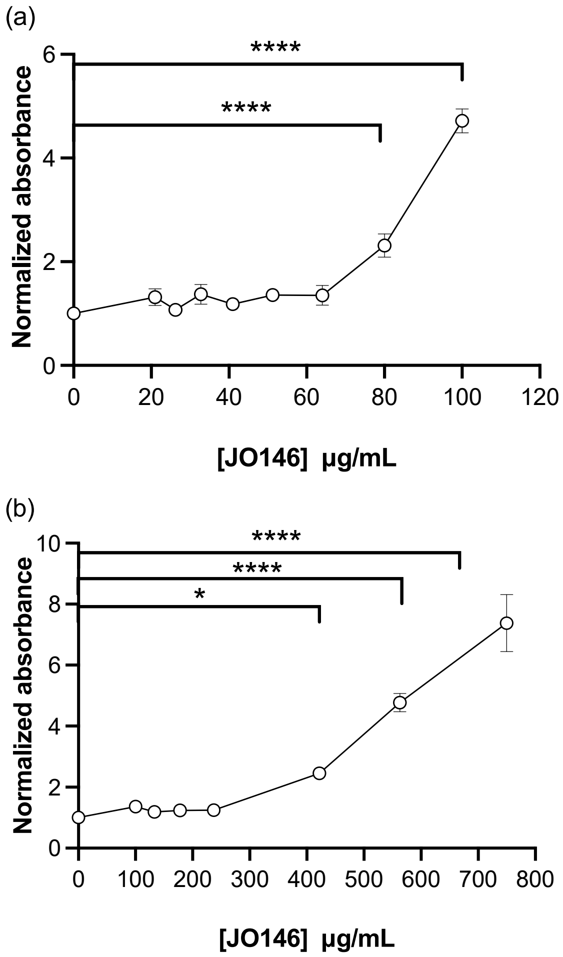

Figure S2. JO146 solubility determination by turbidity assays.

Supplementary Material S7: Survival percentages of *H. pylori* measured by MTT colorimetric assay after administration of free JO146 and a tetracycline control. Survival percentages were obtained by (1) subtracting the raw UV reading of each test well by the average of the negative control wells, then (2) the normalized data were divided by the negative control and multiplied by 100. A <0.1 % survival rate represents a lack of bacterial growth (i.e. 100% growth inhibition) while >100% survival rate indicates that the drug had no effect on the bacterial growth compared to the negative control. Qualitative assessment of MIC was also achieved by the colour change of the MTT tetrazolium salt from yellow to a pink or purple formazan by the naked eye. Each replicate experiment was tested in triplicate hence the average survival rate and SEM (n=3) was reported at each tested concentration. *Estimation of MIC in each replicate experiment.

Table S7. Quantitative and qualitative estimation of MIC of free JO146 by MTT colorimetric assays.

Tetracycline concentration	Average survival (%)	SEM (± %)	Well colour	JO146 concentration	Average survival (%)	SEM (± %)	Well colour
Replicate 1							
5 µg/mL	<0.1	0.77	Yellow	500 µM (300.84 µg/mL)	<0.1	5.83	Yellow
2.5 µg/mL	<0.1	1.16	Yellow	250 µM (150.42 µg/mL)	1.3	1.12	Yellow
1.25 µg/mL*	<0.1	2.36	Yellow	125 µM (75.21 µg/mL)	<0.1	0.82	Yellow
0.63 µg/mL	12.8	6.09	Pink	62.5 µM (37.61 µg/mL)*	<0.1	1.27	Yellow
0.31 µg/mL	41.2	4.62	Purple	31.25 µM (18.80 µg/mL)	22.6	16.26	Pink
0.16 µg/mL	50.8	1.91	Purple	15.63 µM (9.40 µg/mL)	45.6	17.64	Purple
0.08 µg/mL	58.6	3.13	purple	7.81 µM (4.70 µg/mL)	56.8	5.51	purple
0.04 µg/mL	>100	8.39	purple	3.91 µM (2.35 µg/mL)	>100	2.85	purple
Replicate 2							
5 µg/mL	<0.1	1.05	Yellow	500 µM (300.84 µg/mL)	11.7	0.80	Yellow
2.5 µg/mL	<0.1	2.46	Yellow	250 µM (150.42 µg/mL)	8.8	2.61	Yellow
1.25 µg/mL	<0.1	1.28	Yellow	125 µM (75.21 µg/mL)	<0.1	1.71	Yellow
0.63 µg/mL*	<0.1	4.83	Yellow	62.5 µM (37.61 µg/mL)*	<0.1	0.38	Yellow
0.31 µg/mL	33.9	10.78	Pink	31.25 µM (18.80 µg/mL)	27.5	1.56	Pink
0.16 µg/mL	>100	8.96	Purple	15.63 µM (9.40 µg/mL)	33	3.21	Purple
0.08 µg/mL	69.1	0.57	purple	7.81 µM (4.70 µg/mL)	31.4	2.41	purple
0.04 µg/mL	>100	4.71	purple	3.91 µM (2.35 µg/mL)	80.9	0.07	purple
Replicate 3							
5 µg/mL	<0.1	4.06	Yellow	200 µM (120.34 µg/mL)	<0.1	3.66	Yellow
2.5 µg/mL	<0.1	3.73	Yellow	100 µM (60.17 µg/mL)	<0.1	2.86	Yellow
1.25 µg/mL	<0.1	4.97	Yellow	50 µM (30.08 µg/mL)*	<0.1	1.51	Yellow
0.63 µg/mL*	<0.1	4.91	Yellow	25 µM (15.04 µg/mL)	90.2	10.81	Purple
0.31 µg/mL	39.9	15.88	Pink	12.5 µM (7.52 µg/mL)	>100	9.62	Purple
0.16 µg/mL	53	5.21	Purple	6.25 µM (3.76 µg/mL)	>100	1.31	Purple
0.08 µg/mL	73.2	2.31	Purple	3.13 µM (1.88 µg/mL)	>100	1.57	Purple
0.04 µg/mL	63.0	8.7	purple	1.56 µM (0.94 µg/mL)	73	14.95	purple

References

1. Sartorius Stedim Data Analytics AB. User guide to MODDE Version 12. **2017**, doi:https://blog.umetrics.com/hubfs/Download Files/MODDE 12.0.1 User Guide.pdf.
2. Eriksson, L.; Johansson, E.; Kettaneh-Wold, N.; WikstrÄom, C.; Wold, S. *Design of Experiments, Principles and Applications*; Umetrics AB: 2008.
